# Characterizing the pathotype of neonatal meningitis causing *Escherichia coli* (NMEC)

**DOI:** 10.1186/s12866-015-0547-9

**Published:** 2015-10-14

**Authors:** D. S. S. Wijetunge, S. Gongati, C. DebRoy, K. S. Kim, P. O. Couraud, I. A. Romero, B. Weksler, S. Kariyawasam

**Affiliations:** Department of Veterinary and Biomedical Sciences, Pennsylvania State University, 115 Henning Bldg, University Park, Pennsylvania USA; E. coli Reference Center, Department of Veterinary and Biomedical Sciences, Pennsylvania State University, University Park, Pennsylvania USA; Division of Pediatric Infectious Diseases, Johns Hopkins University School of Medicine, Baltimore, Maryland USA; Department of Cell Biology, University of Paris Descartes, Paris, France; Department of Biological Sciences, Open University, Milton Keynes, UK; Department of Medicine, Weill Cornell Medical College in New York, New York, USA; Center for Molecular Immunology and Infectious Disease, Pennsylvania State University, University Park, Pennsylvania USA

**Keywords:** Biofilms, Escherichia coli, Genotyping, Invasion assay, Neonatal meningitis, Serotyping, Virulence

## Abstract

**Background:**

Neonatal meningitis-causing *Escherichia coli* (NMEC) is the predominant Gram-negative bacterial pathogen associated with meningitis in newborn infants. High levels of heterogeneity and diversity have been observed in the repertoire of virulence traits and other characteristics among strains of NMEC making it difficult to define the NMEC pathotype. The objective of the present study was to identify genotypic and phenotypic characteristics of NMEC that can be used to distinguish them from commensal *E. coli.*

**Methods:**

A total of 53 isolates of NMEC obtained from neonates with meningitis and 48 isolates of fecal *E. coli* obtained from healthy individuals (HFEC) were comparatively evaluated using five phenotypic (serotyping, serum bactericidal assay, biofilm assay, antimicorbial susceptibility testing, and in vitro cell invasion assay) and three genotypic (phylogrouping, virulence genotyping, and pulsed-field gel electrophoresis) methods.

**Results:**

A majority (67.92 %) of NMEC belonged to B2 phylogenetic group whereas 59 % of HFEC belonged to groups A and D. Serotyping revealed that the most common O and H types present in NMEC tested were O1 (15 %), O8 (11.3 %), O18 (13.2 %), and H7 (25.3 %). In contrast, none of the HFEC tested belonged to O1 or O18 serogroups. The most common serogroup identified in HFEC was O8 (6.25 %). The virulence genotyping reflected that more than 70 % of NMEC carried *kpsII*, K1, *neuC*, *iucC*, *sitA*, and *vat* genes with only less than 27 % of HFEC possessing these genes. All NMEC and 79 % of HFEC tested were able to invade human cerebral microvascular endothelial cells. No statistically significant difference was observed in the serum resistance phenotype between NMEC and HFEC. The NMEC strains demonstrated a greater ability to form biofilms in Luria Bertani broth medium than did HFEC (79.2 % vs 39.9 %).

**Conclusion:**

The results of our study demonstrated that virulence genotyping and phylogrouping may assist in defining the potential NMEC pathotype.

**Electronic supplementary material:**

The online version of this article (doi:10.1186/s12866-015-0547-9) contains supplementary material, which is available to authorized users.

## Background

*Escherichia coli* is a versatile bacterial species that exists as a commensal in the lower gastrointestinal tract of humans and animals as well as a pathogen that causes a variety of diseases [1, 2]. Unlike commensal *E. coli*, pathogenic *E. coli* harbor various virulence genes which provide the basis for categorizing them into different pathovars with each pathovar having the ability to establish a distinct infection [1]. These virulence traits include adhesins, iron acquisition systems, toxins, invasins, and serum resistant components of the cell wall that are encoded by the bacterial chromosome and plasmids [1–6].

A distinct pathotype of extra-intestinal pathogenic *E. coli* (ExPEC) known as neonatal meningitis-causing *E. coli* (NMEC) have the ability to survive in blood and invade meninges of infants to cause meningitis [1, 7, 8]. *Escherichia coli* associated neonatal meningitis is one of the most common infections that accounts for high mortality and morbidity rates (10–30 %) during the neonatal period [1, 9]. Although distinct sets of virulence traits have been identified in diarrheagenic *E. coli* and other ExPEC pathovars, distinct virulence traits have not been identified to define the NMEC pathotype [10]. Several studies have attempted to characterize NMEC strains using phenotypic and genotyping methods, such as serotyping, multi-locus sequence typing (MLST), phylogrouping, pulsed-field gel electrophoresis (PFGE), antibiotic resistance gene profiling, and virulence genotyping [7, 10–13]. A recent study showed that NMEC strains are diverse in their virulence gene repertoire and the genes that are uniquely associated with NMEC have yet to be identified [10]. Although NMEC have to breach the blood brain barrier (BBB) to reach and infect the meninges, the studies that used various genotypic and phenotypic traits to characterize NMEC have not compared the ability of different NMEC strains to invade the BBB using a model comparable to human brain microvascular endothelial cells. Here, we attempted to determine the genotypic and phenotypic characteristics of NMEC including their ability to invade the BBB and identify a set of virulence genes that were relatively common in NMEC as compared to fecal *E. coli* from healthy humans (HFEC).

## Results

### Phylogenetic typing of NMEC and HFEC

All four major phylogenetic lineages (A, B1, B2, and D) were represented by both NMEC and HFEC although the distribution of each lineage among the isolates belonging to two sources (i.e. NMEC and HFEC) (Table 1, Additional file 1: Table S1) was different. Phylogroup B2 was overly represented in NMEC (67.92 %) as compared to HFEC (29.17 %) (*p* < 0.0001) whereas the phylogroups A and B1 were more common in HFEC (31.25 % and 12.5 %, respectively) than in NMEC (9.43 % and 3.77 %, respectively) (*p* = 0.0001 and *p* = 0.0225, respectively). No statistical difference was observed for the distribution of group D between NMEC and HFEC (*p* = 0.2395).

**Table 1:** Distribution of different phylogroups between neonatal meningitis *E. coli* (NMEC) and fecal *E. coli* from healthy individuals (HFEC). A *p* <0.05 reflects a statistical significance

Strains	Phylogroup (%)			
	A	B1	B2	D
NMEC	9.43	3.77	67.92	18.87
HFEC	31.25	12.5	29.17	27.08
*p* value	0.001	0.0225	<0.001	0.2359

### Serotyping

**Fig. 1:**
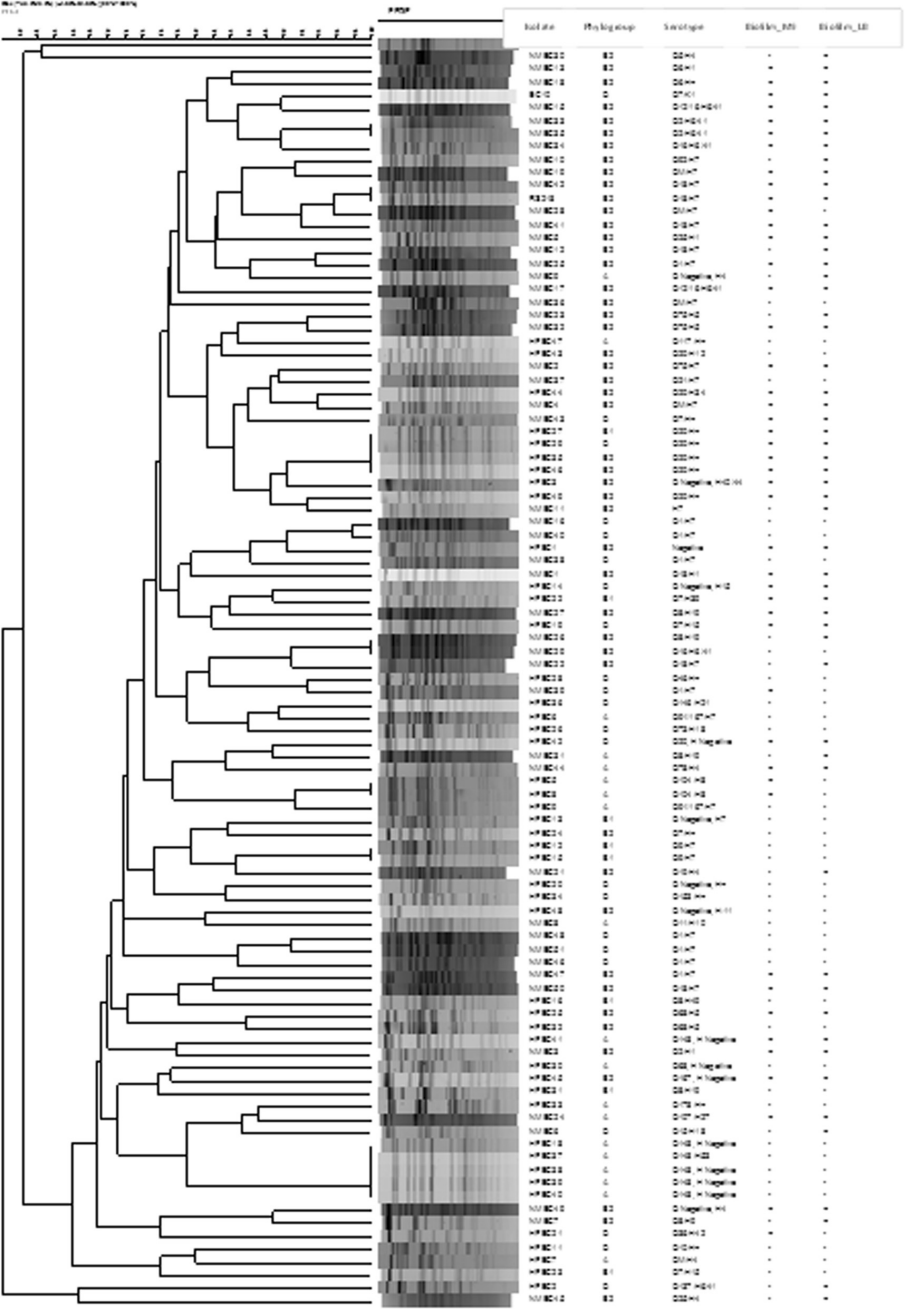
Characteristics of neonatal meningitis *E. coli* (NMEC) and fecal *E. coli* from healthy individuals (HFEC) used in the study

Serotyping of NMEC and HFEC isolates (Fig. 1 and Table 2) showed that 18 different O serogroups were present in NMEC while 19 different O groups were observed in HFEC. Three isolates of NMEC and six isolates of HFEC were not typeable by the O antisera used in the study. Another four NMEC and two HFEC demonstrated multiple O antigen types. The most common O types present in NMEC were O1 (*n* = 9), O8 (*n* = 6), and O18 (*n* = 7) whereas O20 (*n* = 8) and O148 (*n* = 5) were more common in HFEC than the other O types. Similarly, among NMEC strains tested, ten different H types were observed, H7 being the most prevalent (*n* = 24). HFEC strains belonged to 19 different H types with nine isolates demonstrating an H- negative phenotype.

**Table 2:** Distribution of O and H antigen types among NMEC and HFEC strains

NMEC				HFEC			
O Type	No. of Isolates	H Type	No. of Isolates	O Type	No. of Isolates	H Type	No. of Isolates
1	9	1	4	7	4	4	1
2	3	4	6	8	3	5	2
5	1	5	4	9	2	7	5
6	2	7	24	10	1	8	2
7	2	9	1	20	8	11	2
8	6	10	1	36	1	12	1
11	1	18	1	38	1	15	3
15	1	19	3	46	1	18	1
16	2	27	1	68	3	19	1
18	7	6/41	6	73	1	21	1
19	1	POS	2	104	2	34	1
21	1			117	1	39	1
25	2			137	1	42	1
75	3			146	1	49	1
78	1			148	5	53	1
92	1			153	1	6/41	1
107	1			167	2	40/44	1
12/16	2			178	1	NEG	9
M	4			91/167	2	POS	13
NEG	3			M	1		
				NEG	6		

*M* multiple O types, *POS* positive *fliC* PCR amplification but untypeable, *NEG* negative (no agglutination with O antisera or no *fliC* PCR amplification)

### Serum resistance

No statistically significant difference in the percentages of serum resistant isolates between NMEC and HFEC (91.82 ± 2.17 and 90.97 ± 1.2, respectively) was observed (*p* =0.5172).

### Virulence genotyping

**Fig. 2:**
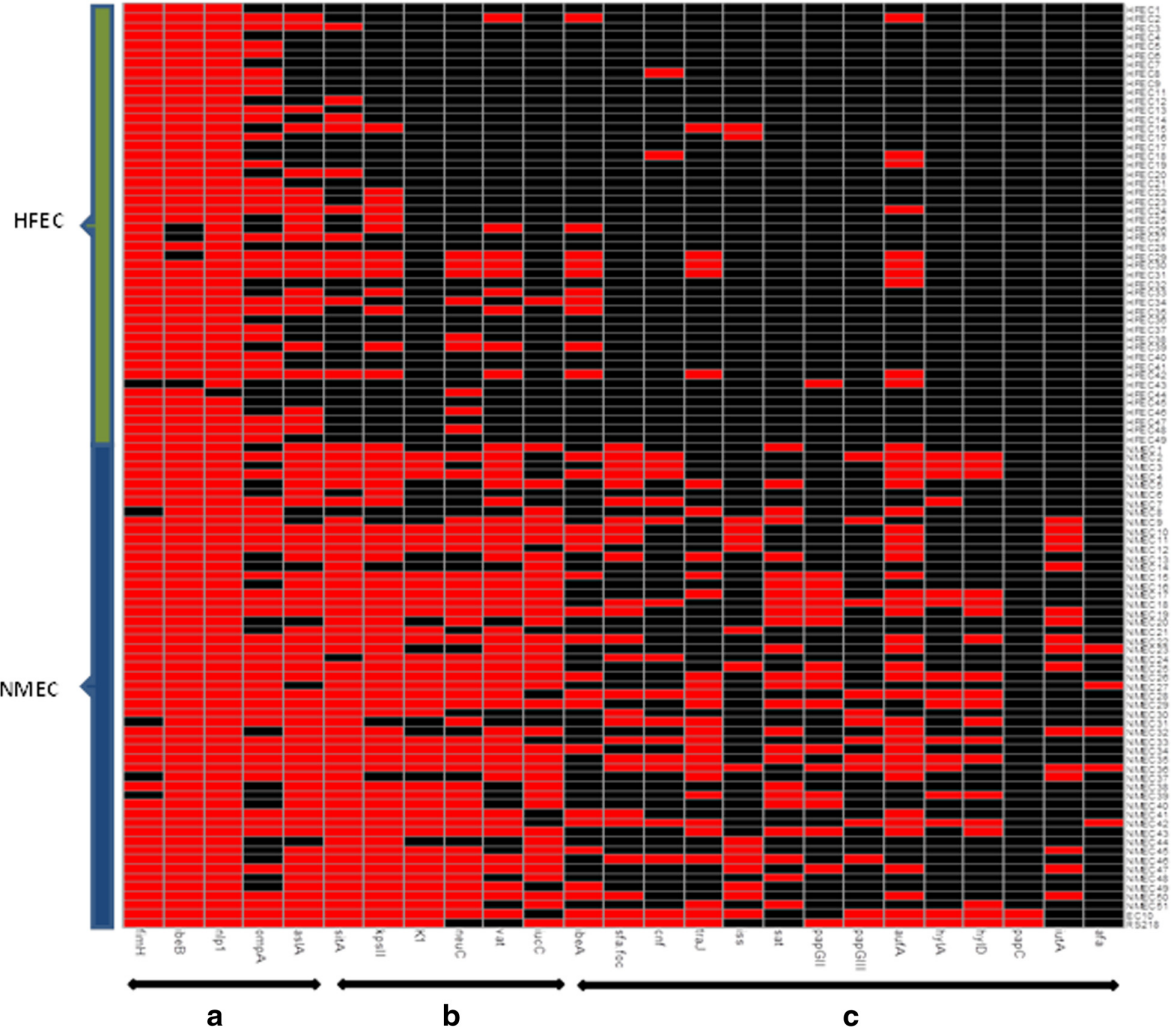
Heat map demostrating the distribution of virulence genes in NMEC and HFEC. Red represents the presence and black represents the absence of a virulence gene; **a**, virulence genes equally present in both NMEC and HFEC; **b**, virulence genes are more prevalent and overly represented in NMEC than in HFEC; **c**, virulence genes are less prevalent in both NMEC and HFEC

The number of virulence traits present in NMEC was higher (13 ± 3.84) than in HFEC (5.50 ± 2.49) revealing a considerable variation between the virulence gene profiles of *E. coli* from two different sources (Additional file 1: Table S1). For example, of the 26 VFs examined in the study, the occurrence of 21 genes was significantly higher (*p* <0.05) in NMEC than in HFEC (Fig. 2 and Table 3). Notably, K1 capsular type*, sfa/foc*, *sat*, *hlyA*/*D*, *iutA*, *papG III*, and *afa* genes were only represented in NMEC populations, although their occurrence among NMEC isolates was low except for the K1 capsular type. Virulence traits *kpsII*, *sitA*, *neuC*, i*ucC*, and *vat* were more commonly associated with NMEC than with HFEC (prevalence >70 % and representation >2-folds higher in NMEC than in HFEC). Similar to the observations made by previous studies, some virulence genes that were implicated in the penetration of BBB, such as *fimH*, *npl*, and *ibeB* were equally present in both NMEC and HFEC strains [10]. On the other hand, some virulence genes, such as *cnf1* and *traJ*, that were considered to be required for NMEC pathogenesis, were possessed only by a minority of NMEC (*cnf1* and *traJ* in 27.45 % and 43.40 % of NMEC, respectively). However, these genes were more frequently observed in NMEC than in HFEC (Table 3).

**Table 3:** Occurrence (%) of virulence genes between neonatal meningitis *E. coli* (NMEC) and fecal *E. coli* (HFEC)

Gene	NMEC	HFEC	Chi Square value	*p* value
*afa*	9.43	0.00	7.446	0.0064
*aslA*	88.68	45.83	41.814	<0.0001
*aufA*	62.26	20.83	32.952	<0.0001
*cnf*	27.45	4.17	20.19	<0.0001
*fimH*	92.45	100.00	6.380	0.0115
*hly*	30.19	0.00	32.980	<0.0001
*hlyD*	35.85	0.00	45.995	<0.0001
*ibeA*	35.85	20.83	6.881	0.0087
*ibeB*	100.00	91.67	6.380	0.0115
*iss*	25.49	4.17	16.132	<0.0001
*iucC*	**74.51**	**2.08**	**109.471**	**<0.0001**
*iutA*	30.19	0.00	32.980	<0.0001
K1	**75.47**	**0.00**	**116.821**	**<0.0001**
*kpsII*	**90.57**	**27.08**	**82.038**	**<0.0001**
*neuC*	**71.70**	**18.75**	**54.522**	**<0.0001**
*nlp1*	100.00	97.92	0.505	0.4773
*ompA*	66.04	60.41	0.536	0.464
*papC*	3.77	0.00	2.296	0.129
*papGII*	31.37	2.08	25.833	<0.0001
*papGIII*	21.57	0.00	22.523	<0.0001
*sat*	49.02	0.00	62.279	<0.0001
*sfa/foc*	49.06	0.00	62.279	<0.0001
*sitA*	**92.45**	**25.00**	**89.713**	**<0.0001**
*traJ*	43.40	10.41	26.287	<0.0001
*vat*	**76.47**	**18.75**	**32.829**	**<0.0001**

*p* <0.05 reflects a statistical significance. Virulence genes that were more prevalent in NMEC compared to HFEC are marked in bold

### PFGE

Out of the NMEC and HFEC isolates tested, only 98 were typeable by PFGE. The HFEC strain 17 and two NMEC isolates (14 and 20) were nontypeable with *Xba*I restriction digestion. The typeable isolates demonstrated 86 distinct PFGE fingerprint patterns or pulsotypes (Fig. 1). Of the 86 pulsotypes, 79 were unique and each pattern was present only in one isolate. The other seven pulsotypes were present in two or more isolates of *E. coli*. Not a single pulsotype was shared between NMEC and HFEC.

### Biofilm assay

The ability to form biofilms on a microtitre plate by NMEC and HFEC were assessed using M9 minimal medium supplemented with niacin and Luria Bertani (LB) broth (Additional file 1: Table S1). About 53 % of NMEC strains cultured in M9 minimal medium were able to form biofilms on microtitre plates whereas only 39.9 % of HFEC possessed this phenotype (Fig. 1). However, this difference was not statistically significant (*p* = 1.000). A higher percentage of NMEC (79.2 %) formed biofilms when they were cultured in LB broth than in the M9 medium (52.8 %) but the ability to form biofilms by HEFC remained the same regardless of the medium used.

### Antibiotic susceptibility testing and multilocus sequence typing (MLST)

As depicted in Table 4, a majority of NMEC and HFEC were susceptible to most of the antimicrobials tested in the study. All isolates tested were susceptible to gentamicin, imipenam, meropenem, piperacillin/tazobactam and amikacin. Only five isolates of NMEC and three isolates of HFEC were resistant to all four third generation cephalosporins (cefotaxime, cefpodooxime, ceftazidine and ceftrixone). The minimal inhibitory concentration (MIC) values for these eight isolates were increased by ≥3 folds when cefotaxime and ceftazidine were used in combination with clavulanic acid as compared to the MIC values for cefotaxime and ceftazidine alone confirming that these isolates possessed the ESBL resistance phenotype according to the Clinical Laboratory Standards Institute (CLSI) guidelines. To further characterize *E. coli* demonstrating an ESBL phenotype, they were subjected to MLST. The eight isolates with an ESBL phenotype fell into eight different sequence types (STs) demonstrating a clonal diversity among the isolates (Table 5).

**Table 4:** Antibiotic susceptibility profiles of NMEC and HFEC. Number of isolates (stated as percentages) in NMEC and HFEC were categorized as Sensitive (S), Intermediate (I) or Resistant (R) according to CLSI standards

Antibiotic	NMEC			HFEC		
	S	I	R	S	I	R
Ampicillin	50.94	3.773585	45.28	64.58	4.167	31.25
Cefazolin	96.23	0	3.77	93.75	0	6.25
Cefepime	96.23	0	3.77	100	0	0
Cefotaxime	90.57	0	9.43	93.75	0	6.25
Cefoxitin	90.57	0	9.43	95.83	2.08	2.08
Cefpodoxime	90.57	0	9.43	9.16	0	8.33
Ceftazidime	90.57	0	9.43	93.75	0	6.25
Ceftriaxone	88.68	1	9.43	91.66	2.08	6.25
Cephalothin	100	0	0	100	0	1
Ciprofloxacin	100	0	0	100	0	0
Gentamicin	100	0	0	100	0	0
Imipenem	100	0	0	100	0	0
Meropenem	100	0	0	100	0	0
Piperacillin/Tazobactam	100	0	0	100	0	0
Ceftazidime/Clavulanic acid	100	0	0	100	0	0
Amikacin	100	0	0	100	0	0
Amoxicillin	100	0	0	60.41	2.083	37.5
Chloramphenicol	94.34	0	5.66	97.91	2.083	0
Sulfa/Trimethoprim	81.13	0	18.86	93.75	0	6.25
Kanamycin	86.79	2	9.433	100	0	0
Nalidixic acid	96.23	0	3.773	81.25	0	18.75
Sulfisoxazole	67.92	3	14	68.75	16.66	14.58
Tetracycline	81.13	0	10	91.66	0	8.33

**Table 5:** Serotypes and multilocus sequence types (ST) of ESBL-positive isolates

Isolate	Serotype	Multilocus sequence alleles							ST
		*adk*	*fumC*	*gyrB*	*icd*	*mdh*	*purA*	*recA*	
NMEC2	O75:H7	40	20	19	14	23	1	10	ST843
NMEC3	O2:H1	36	24	9	13	17	11	25	ST73
NMEC44	O78:H4	6	4	12	1	20	13	7	ST23
NMEC46	O1:H7	27	32	24	29	26	19	22	ST59
NMEC50	O18:H7	37	38	19	37	17	11	26	ST95
HFEC2	O137:H6/41	13	14	19	22	17	14	203	ST2678
HFEC9	O167:H7	6	4	5	18		8	14	Unknown ST
HFEC13^a^	H7	6	19	3	16	11	8	173	Unknown ST

^a^No agglutination with O antisera

### In-vitro cell invasion assay

**Fig. 3:**
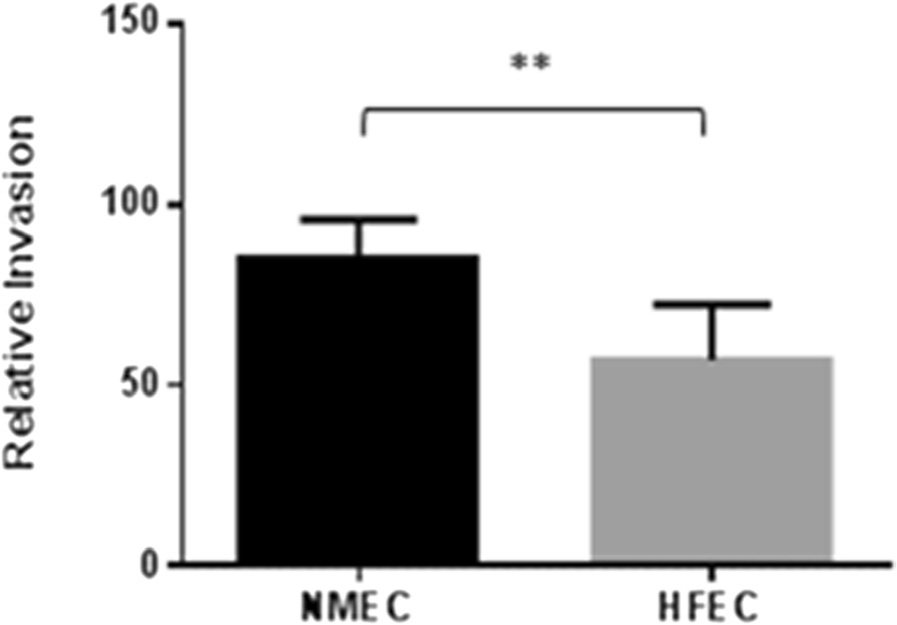
Relative invasion frequencies observed between NMEC and HFEC populations. **Denotes a statistically significant difference (*p* < 0.05)

Invasion frequencies between NMEC and HFEC (85.31 ± 10.60 vs 54.04 ± 7.0387, respectively; *p* = 0.0196) displayed a statistically significant difference (Fig. 3). Except ten isolates of HEFC, all other HFEC and NMEC isolates were able to invade human cerebral microvascular endothelial cells (hCMECs) albeit at different invasion frequencies (Additional file 1: Table S1). Some HFEC strains demonstrated invasion frequencies similar to or higher than that of the prototypic NMEC strain RS218.

## Discussion

Pathogenic *E. coli* consist of diarrheagenic *E. coli* causing intestinal disease and ExPEC causing extraintestinal diseases in humans and animals [1, 2]. Both diarrheagenic *E. coli* and ExPEC are grouped into many pathotypes or pathovars depending on the clinical outcome of the disease and virulence properties of *E. coli* involved [1, 11, 14]. Of these two major groups of pathogenic *E. coli,* ExPEC possess the ability to colonize and infect extraintestinal sites of the host by means of horizontally acquired genetic determinants or virulence genes [14]. Several studies have been conducted to identify the unique virulence traits of ExPEC, such as uropathogenic *E. coli*, NMEC, and avian pathogenic *E. coli* to understand the genetic makeup and pathogenic potential of different ExPEC pathovars [10–13]. However, these studies indicate that substantial genotypic and phenotypic heterogeneity exist within each pathotype, and a pathotype cannot be defined merely based on the presence of a defined set of virulence genes or characteristics. Nevertheless, some virulence attributes are shared among different pathotypes making it difficult to delineate ExPEC pathotypes [7, 10, 12, 13, 15]. Here, we comparatively analyzed genotypic and phenotypic traits of NMEC and HFEC to identify the characteristics that would assist in defining the NMEC pathotype.

**Fig. 4:**
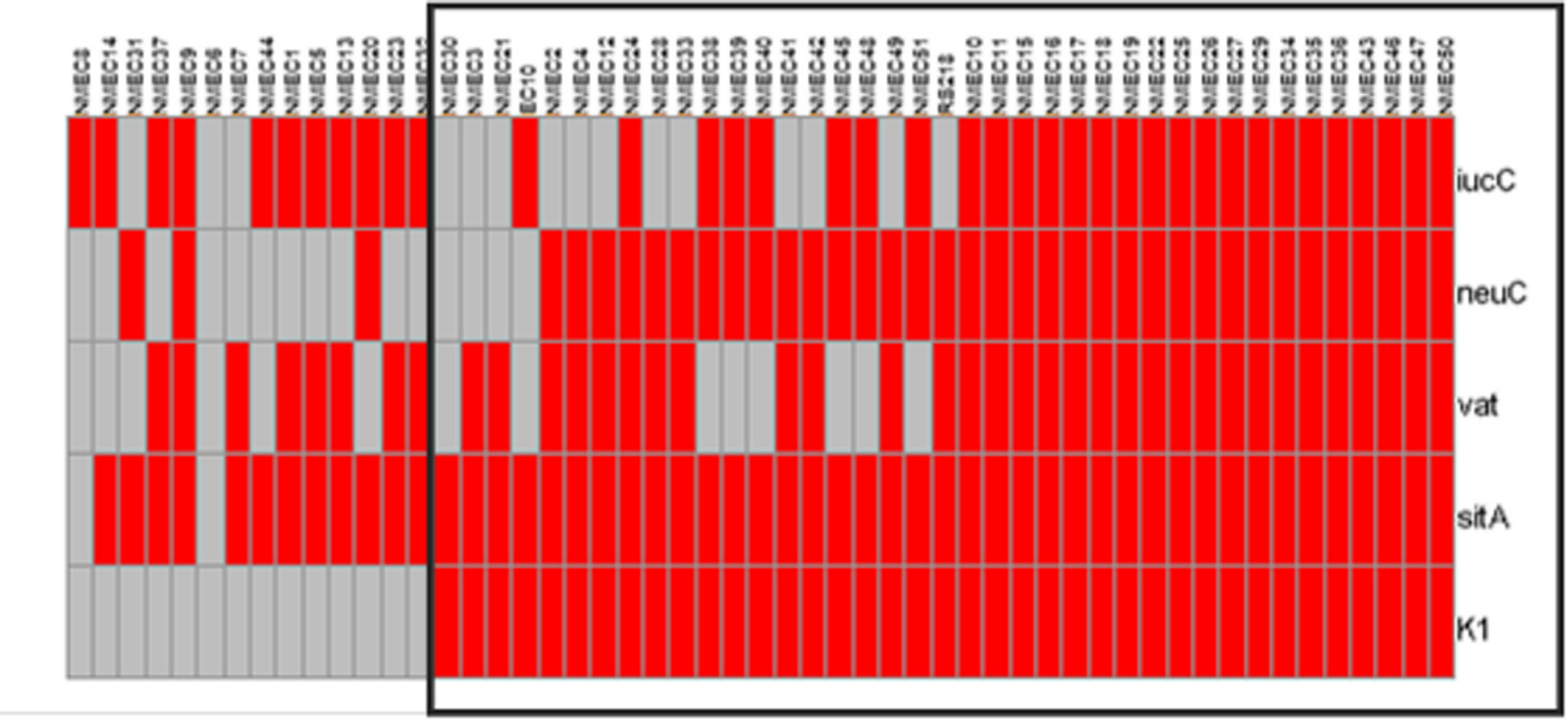
Distributin of K1, *sitA*, *vat*, *neuC*, and *iucC* genes among NMEC strains (*n* = 53). Red, positive for a gene; Grey, negative for a gene. Encircled area indicates the isolates used to define a typical NMEC

In this study, we examined a collection of NMEC for the most prevalent (>70 % of the NMEC population) and overly represented virulence traits found in NMEC as compared to HFEC (>3-fold) for defining the NMEC pathotype. Based on these criteria, we have identified six genes, namely, *kpsII*, K1, *neuC*, *iucC*, *sitA,* and *vat*, which were predominantly associated with NMEC pathotype. Among these, some virulence traits such as, *kpsII*, *sitA*, and *vat* are considered as typical traits of NMEC [10]. None of the HFEC isolates surveyed possessed all six genes, whereas 40 % of NMEC isolates harbored all six genes suggesting that these genes might be used to predict the NMEC pathotype (Fig. 4). As depicted in Fig. 4, a majority of NMEC possessed the K1 capsular type (*n* = 76 %). All K1^+^ NMEC were positive for *sitA* together with *vat* and/or *iucC.* Except three isolates, all other K1^+^ NMEC harbored the *neuC* gene as well. Taken together, these results suggest that a typical NMEC might be described as K1^+^ and *sitA*^+^ and having at least two of the other three genes; *vat*, *neuC* and *iucC*. Using this criterion, it was possible to designate 74 % of NMEC used in this study as typical NMEC whereas none of the HFEC satisfied these benchmarks. However, typing of a larger collection of NMEC isolates representing different geographical regions might be required to confirm these observations.

A PCR-based phylogrouping has been established to determine the clonality within *E. coli* pathotypes [16, 17]. Previous studies have identified group B2 as the most common phylogroup of ExPEC suggesting that phylogrouping may be used as a rapid typing method to identify potential ExPEC [10, 12, 14, 18]. In the present study, we observed that 67.9 % of NMEC fell into B2 phylogroup as compared to 29.2 % of HFEC tested in the study. Phylogroups A and B1 were more commonly associated with HFEC than with NMEC. The virulence gene profiles of NMEC strains belonging to each phylogroup lineage exhibited a higher prevalence of virulence genes as compared to HFEC strains belonging to the corresponding lineage (Table 6). Taken together, our observations emphasize that phylogrouping alone is not suitable for predicting potential NMEC.

**Table 6:** Number of virulence traits present in neonatal meningitis *E. coli* (NMEC) and fecal *E. coli* (HFEC) belonging to different phylogroups

Phylogroup	No. Virulence associated traits (Mean ± SD)		*p* value
	NMEC	HFEC	
A	10 ± 2.74	5.13 ± 2.33	0.0011^a^
B1	8.0 ± 2.83	6.5 ± 3.02	0.5610
B2	15.19 ± 3.21	4.64 ± 1.95	<0.0001^a^
D	12.4 ± 3.92	6.38 ± 2.79	0.0003^a^

^a^Denotes a statistically significant difference between NMEC and HFEC

Pulsotyping and serotyping have been used in various studies to identify pathogenic clones and to distinguish between pathogenic and nonpathogenic strains of many bacterial species [7, 19, 20]. In our study, we observed a considerable heterogeneity among both NMEC and HFEC. As depicted in Table 2, despite this diversity, many NMEC strains belonged to O1, O8, and O18 serogroups and H7 flagellar antigen type. Similar to our observation, previous studies have also noted that the H7 antigen is present in major virulent clones of ExPEC pathotypes making it a potential target for future diagnostic and therapeutic interventions [21, 22]. Two subtypes of H7 antigen have been identified for enterohemorrhagic *E. coli* (EHEC) based on the *flicC* (H7) allelic polymorphism, a technique which possibly can be used to type EHEC [23, 24]. For example, H7a and H7c subtypes have been identified in *E. coli* O157: H7 irrespective of their geographical origin and genetic variation observed with other molecular typing methods [23]. However, whether this genetic polymorphism of H7 does or does not exist in ExPEC pathotypes is currently unknown. Future studies focused on H7 allelic polymorphism of ExPEC may provide useful information to develop a novel subtyping technique to distinguish different ExPEC pathovars. The heterogeneity observed with PFGE was even greater than serotyping indicating that PFGE is not useful in distinguishing NMEC strains from HFEC.

Ability to form biofilms in different environmental conditions facilitates bacterial survival on abiotic surfaces and their resistance to antimicrobials and antiseptics [25, 26]. Given that biofilm formation is reported to assist in bacterial survival, colonization, and protection from host immune responses and antimicrobial agents, the ability to produce biofilms may play an important role in NMEC virulence [25–27]. Therefore, we compared the capacity for biofilm formation by NMEC and HFEC. There was no significant difference in the ability of biofilm formation between NMEC and HFEC isolates when they were grown in M9 medium. However, when LB broth was used as the medium, NMEC demonstrated approximately 39 % increase in their ability to form biofilms. In consistent with our observations, previous studies have demonstrated that growth medium has an effect on biofilm formation by pathogenic bacteria [28, 29] . Jackson *et al.* noted that central carbon flux has a regulatory effect on the formation of biofilms [28]. In particular, readily available glucose has a negative regulatory effect on biofilm formation suggesting that the ability to form biofilms is a survival strategy by bacteria when the source of carbon is limited or complex [28]. The M9 medium contained 2 % glucose which is a readily available carbon source whereas in LB broth catabolizable amino acids are the source carbon source for bacterial growth. Increase in biofilm formation by NMEC in LB broth as compared to M9 medium might be due to readily available glucose in M9 medium suggesting that the source of carbon has an effect on biofilm formation by NMEC but not by HFEC.

Beta-lactam antibiotics are one of the major treatment options available for controlling meningitis in infant patients [30]. We evaluated the antibiotic sensitivity profiles of NMEC and HFEC along with ESBL resistance profiles. Although the majority of strains were sensitive to most of the antibiotics tested, eight isolates showed an ESBL-resistance phenotype. Interestingly, three of the ESBL-resistant isolates were fecal commensal isolates implying fecal flora as a source for the emergence of ESBL-resistance. Previous studies have indicated an association between ESBL types and MLST types [31]. The ESBL-positive *E. coli* identified in this study fell into ST73, ST23, and ST59 MLST types, which are known to contain *E. coli* possessing certain ESBL types. However, none of the ESBL-positive *E. coli* detected here did belong to ST131, a major MLST type associated with ESBL-positive *E. coli* [31]. The ESBL genes are commonly located on mobilizable elements (e g. transposons and broad host range plasmids) and ESBL genes are distributed among commensal microflora of humans and animals [32]. Although a positive correlation between the presence of ESBL-positive HFEC and ESBL-positive UPEC has been observed by many investigators, there is no evidence to suggest an association between the presence of ESBL-producing bacteria in vaginal or fecal flora of pregnant women and corresponding ESBL-producing bacterial infections in neonates [33]. Nevertheless, it has been demonstrated that the risk factors, such as premature delivery, premature membrane rupture, and low birth weight which are known to predispose infants to *E. coli* meningitis were correlated with the colonization of ESBL-producing *Enterobacteriaceae* in the infant gut [33].

Genetic heterogeneity of NMEC pathotype is one of the major constraints posing today to understand the pathogenesis of neonatal meningitis [7, 10, 12]. Concordance with these previous studies, we have also observed marked heterogeneity in the virulence gene profiles of NMEC. Although NMEC possessed more virulence genes than HFEC, some of the virulence genes (*ibeA*, *sfa/foc*, *traJ,* and *cnf1*), which have previously been implicated in NMEC pathogenesis were present only in <50 % of NMEC examined (Fig. 2) [34–36]. The virulence traits, such as *fimH*, *ompA,* and *nlp1* that are considered to be essential for NMEC survival in blood and the penetration of BBB were prevalent in both NMEC and HFEC suggesting these genes do not define the NMEC pathotype [34–39]. The NMEC and HFEC strains that did not harbor these “essential” virulence genes were still able to invade hCMEC/D3. These data indicate that NMEC pathogenicity is poorly understood and highlight the need for future research directed at mining novel virulence traits that are truly involved in the penetration of BBB.

In the present study, NMEC strains harbored genes that encode iron acquisition systems (*iutA*, *iucC,* and *sitA*) and hemolysins (*hlyA* and *hlyD*), which were more frequently present in NMEC than in HFEC. In addition to providing a source of iron for bacterial growth through cell lysis, bacterial hemolysins are known to play some other mechanistic roles as well [40]. For example, sublytic amounts of *E. coli* hemolysin have been shown to increase the permeability of endothelial cell monolayers in a time- and dose-dependent manner in cultured pulmonary artery endothelial cells [40] . In the same study, a low dose of hemolysin was found to induce a toxin-mediated loss of endothelial barrier function. However, there is no scientific evidence to support the involvement of hemolysins of NMEC in increasing the permeability of BBB thereby aiding the disease pathogenesis.

In this study, an in vitro invasion assay was conducted to correlate the virulence traits of each isolate of *E. coli* with its ability to penetrate the BBB. As expected, NMEC strains which carried more virulence genes than HFEC were also more invasive in hCMEC/D3 cells than HFEC (Fig. 3). Interestingly, ten isolates of HFEC which demonstrated a noninvasive phenotype with in vitro cell invasion assay were negative for K1 capsular type, *ibeA*, *traJ,* and *sfa/foc* but were positive for *fimH, ibeB,* and *nlpl* indicating that *ibeA*, *traJ,* and *sfa/foc* may be important in NMEC pathogenesis even though their occurrence in NMEC was less than some other virulence genes. Except these ten noninvasive isolates, all other HFEC were able to invade the BBB barrier in vitro suggesting fecal commensal *E. coli* as a potential source of NMEC. It is known that a high level of bacteremia is a prerequisite for the attachment and invasion of BBB by NMEC [9, 41]. The present study also demonstrated that NMEC and HFEC were equally able to survive in human serum, supporting the speculation that commensal *E. coli* in the mother’s gut could be a source for NMEC in the baby. Perhaps serum resistance of NMEC may not be as important as previously thought because neonatal serum is deficient in many complement factors. It would be worthy to compare the survival potential of NMEC in serum collected from healthy adults and neonates. Further investigations on HFEC strains exhibiting invasive properties using animal models of meningitis may be needed to confirm that HFEC indeed have the potential to cause neonatal meningitis.

## Conclusions

The current study results indicate that regardless of the genotypic and phenotypic heterogeneity observed among NMEC strains, some genotypic characteristics can still be used to distinguish NMEC from commensal *E. coli*.

## Materials and methods

### Bacterial strains and media

The NMEC comprised of 51 strains isolated from the cerebrospinal fluid of neonates with meningitis plus two well-characterized NMEC strains, RS218 (O18:K1:H7) and EC10 (O7: K1). The RS218 [42] and EC10 [43, 44] strains isolated from the cerebrospinal fluid of neonates with meningitis in the 1970’s were kindly provided by Dr. James Johnson, University of Minnesota, St. Paul, MN, USA and Dr. David Klumpp, Northwestern University, Evanston, IL, USA, respectively. Other NMEC strains (*n* = 51) isolated between 1989 and 1997 from patients in the United States were provided by Dr. K. S. Kim, Johns Hopkins University School of Medicine, Baltimore, Maryland, USA [10, 42]. Fecal *E. coli* (*n* = 48) isolated from feces of healthy individuals (HFEC) were obtained from the E. coli Reference Center collection (Pennsylvania State University, University, Park, PA, USA). These isolates were collected during the period between 1985 and 2005 from healthy individuals in two states of the U.S., New York and Pennsylvania. Unless otherwise mentioned, all the bacterial strains were grown in Luria Bertani (LB) broth (BD Technologies, Research Triangle Park, NC, USA) or LB agar (BD Technologies).

### Phylogrouping of *E. coli*

All strains were assigned to four main phylogenetic groups; A, B1, B2 and D according to the method described by Clemont *et al.* [17]. Classification of phylogenetic groups was based on the amplification of two genes, *chuA* and *yjaA* and the DNA fragment, TspE4C2 by using a multiplex polymerase chain reaction (PCR) assay. The primers used are listed in Table 7.

**Table 7:** Primers used in the study

Primer Name	Gene/Function	Sequence 5’–3’	Product size	Reference
chuA.1	Phylogrouping	GACGAACCAACGGTCAGGAT	279	[17]
chuA.2		TGCCGCCAGTACCAAAGACA		
yjaA.1	Phylogrouping	TGAAGTGTCAGGAGACGCTG	211	[17]
yjaA.2		ATGGAGAATGCGTTCCTCAAC		
TspE4C2.1	Phylogrouping	GAGTAATGTCGGGGCATTCA	152	[17]
TspE4C2.2		CGCGCCAACAAAGTATTACG		
afaF	Afimbrial adhesin	CAT CAA GCT GTT TGT TCG TCC GCC G	750	[52]
afaR		GCT GGG CAG CAA ACT GAT AAC TCT C		
aslAF	Arylsulfatase	GCG TGA TGT TCA TGT CAA CC	463	[44]
aslAR		ATC CGC CAG ATC TAC AAT GC		
aufAF	Auf fimbriae	TGC ACA TCA GGA AAC CAG ATA C	350	This study
aufR		GGC TCA CTG ATA TGG ATG ACA A		
cnfF	Cytotoxic necrotizing factor1	CAT TCA GAG TCC TGC CCT CAT TAT T	498	[53]
cnfR		AAG ATG GAG TTT CCT ATG CAG GAG		
fimHF	Type I fimbriae	GCA GTC ACC TGC CCT CCG GTA	508	[53]
fimHR		TGC AGA ACG GAT AAG CCG TGG		
hlyAF	Alpha hemolysin	ACC ATA TAA GCG GTC ATT CCC GTC A	1177	[53]
hlyAR		AAC AAG GAT AAG CAC TGT TCT GGC T		
hlyDF	Secretion protein for HlyA	CTC CGG TAC GTG AAA AGG AC	904	[54]
hlyDR		GCC CTG ATT ACT GAA GCC TG		
ibeAF	Invasin of brain endothelium A	CAT TAG CTC TCG GTT CAC GCT	171	[55]
ibeAR		TTA CCG CCG TTG ATG TTA TCA		
ibeBF	Invasin of brain endothelium B	GCA TATTCTGCTGGTTTCTAATGTC	660	This study
ibeBR		GTT CTG GAT TTT CTG TTC ATA ATT CA		
issF	Increased serum survival	CAG CAA CCC GAA CCA CTT GAT G	323	[54]
issR		AGC ATT GCC AGA GCG GCA GAA		
iutAF	Aerobactin synthesis	ATGAGCATATCTCCGGACG	587	[52]
iutAR		CAGGTCGAAGAACATCTGG		
iucCF	Aerobactin synthesis	ACC CGT CTG CAA ATC ATG GAT	269	[55]
iucCR		AAA CCT GGC TTA CGC AAC TGT		
sitAF	Periplasmic iron-binding protein	AGG GGG CAC AAC TGA TTC TCG	608	[56]
sitAR		TAC CGG GCC GTT TTC TGT GC		
kpsMTII F	Capsular type II	GCG CAT TTG CTG ATA CTG TTG	272	[57]
kpsMT IIR		CAT CCA GAC GAT AAG CAT GAG CA		
kpsMT K1	K 1 capsule	TAG CAA ACG TTC TAT TGG TGC	153	[57]
kpsMT IIR		CAT CCA GAC GAT AAG CAT GAG CA		[57]
neuCF	Capsular N-acetylneuraminic acid synthesis	GGT GGT ACA TTC CGG GAT GTC	676	[52]
neuCR		AGG TGA AAA GCC TGG TAG TGT G		
Nlp I F	New lipoprotein I (adhesin/invasin)	AGT AAT ACT TCC TGG CGT AAA AGT GA	662	This study
Nlp I R		AAA TAG AAG TTG GTT TCA CTG AGA TG		
ompAF	Outer membrane protein A	CACTAAATCCAACGTTTATGGTAAAA		This study
ompAR		GATACCTTTAACTTCGATCTCTACGC		
papCF	Outer membrane usher protein of P pili	ATA TCC TTT CTG CAG GGA TGC AAT A	205	[57]
papCR		GAC GGC TGT ACT GCA GGG TGT GGC G		
papG IF	Fimbrial adhesin PapG allele I	CTA CTA TAG TTC ATG CTC AGG TC	474	[54]
papG IR		CCT GCA TCC TCC ACC ATT ATC GA		
papG IIF	Fimbrial adhesin PapG allele II	CCC AGC TTT GTT ATT TTC CTT G	190	[54]
papGIIR		TTC TTA CCA TGG CTG TAT GTC G		
papGIIIF	Fimbrial adhesin PapG allele III	GGC CTG CAA TGG ATT TAC CTG G	258	[54]
papGIIIR		CCA CCA AAT GAC CAT GCC AGA C		
satF	Secreted autotransporter toxin	GCAGCAAATATTGATATATCA	630	[58]
satR		GTTGTTGACCTCAGCAAGGAA		
sfa/focF	S fimbrial adhesin	CGG AGG AGT AAT TAC AAA CCT GGC A	410	[57]
sfa/focR		CTC CGG AGA ACT GGG TGC ATC TTA C		
trajF	Conjugal transfer	CAA TGG GGC TTT TAT TGA ACT C	369	This study
trajR		TGA CCA ACA CCC AGC ATA TAA A		
vat1	Vacuolating cytotoxin	TCC TGG GAC ATA ATG GTC AG	900	This study
vat2		GTG TCA GAA CGG AAT TGT		
adkF	MLST	ATTCTGCTTGGCGCTCCGGG	583	[49]
adkR		CCGTCAACTTTCGCGTATTT		
fumCF	MLST	TCACAGGTCGCCAGCGCTTC	806	[49]
fumR		GTACGCAGCGAAAAAGATT		
gyrBF	MLST	TCGGCGACACGGATGACGGC	911	[49]
gyrBR		GTCCATGTAGGCGTTCAGGG		
icdF	MLST	ATGGAAAGTAAAGTAGTTGTTCCGGCACA	878	[49]
icdR		GGACGCAGCAGGATCTGTT		
mdhF	MLST	ATGAAAGTCGCAGTCCTCGGCGCTGCTGGCGG	932	[49]
mdhR		TTAACGAACTCCTGCCCCAGAGCGATATCTTTCTT		
purAF	MLST	TCGGTAACGGTGTTGTGCTG	816	[49]
purAR		CATACGGTAAGCCACGCAGA		
recAF	MLST	CGCATTCGCTTTACCCTGACC	780	[49]
recAR		TCGTCGAAATCTACGGACCGGA		

### Serotyping

Serotyping was performed at the *E. coli* Reference Center (Pennsylvania State University) using a standard serum agglutination assay for the presence of all designated O groups (O1 to O187 except O13, O22, O31, O47 and O94 that are not designated) and H types (H1 to H56) [45].

### Serum resistance

Survival of bacterial strains (both NMEC and HFEC) in normal human serum (EMD Millipore Corporation, Temecula, CA, USA) was determined by colorimetric microassay as previously described [46]. Briefly, log phase cultures of bacterial strains were incubated for 3 h at 42 °C in Peptone/Glucose broth containing 0.75 % bromothymol blue with or without 20 % normal human serum (NHS). Serum resistance of each strain was determined by comparing the color change from green to yellow in the presence of NHS.

### Virulence typing of *E. coli*

NMEC and HFEC strains were examined for the presence of 26 genes encoding virulence factors (VFs) that have been previously attributed to the establishment of meningitis in neonates [1, 9]. The VFs included adhesins, invasins, toxins, siderophores, and structural components of *E. coli*. Virulence genes were detected by PCR amplification carried out on a Master Cycler Pro (Eppendorf, Hamburg, Germany). All primers were obtained from Integrated DNA Technologies (Coralville, IA, USA) and are listed in Table 7. Crude DNA was extracted by a rapid boiling method [47]. All PCRs were performed in 25-μl reactions containing 1 U of *Taq* DNA polymerase (Denville Scientific Inc., Metuchen, NJ, USA), 25 pmol each of the forward and reverse primers, and 5 nmol of each deoxynucleoside triphosphate (Denville) in 1× buffer (15 mM MgCl_2_, 100 mM KCl, 80 mM (NH_4_)_2_SO_4_, 100 mM Tris–HCl, pH 9.0, 0.5 % NP-40). The cycling conditions were as follows: initial denaturation at 94 °C for 3 min, followed by 30 cycles of three steps consisting denaturation at 94 °C for 1 min, primer annealing at the temperatures indicated in Table 7 for 1 min, and extension at 72 °C for at least 30 s, according to the size of the amplified fragment (1 min/Kbp), and followed by a final extension at 72 °C for 10 min. Products were electrophoresed in a 1.5 % agarose gel (Denville) for 1 h at 120 V, stained with ethidium bromide (Bio-Rad Laboratories, Hercules, CA, USA), and photographed under UV light using a gel documentation system (AlphaImager® HP, Alpha Innotech Corporation, San Leandro, CA, USA). Each PCR included a negative control that contained all reagents except template DNA and a positive control that contained an *E. coli* that is known to carry the respective virulence gene.

### Pulsed-field gel electrophoresis

PFGE was conducted according to the method described by PulseNet [48]. Chromosomal DNA was digested with the *Xba*I restriction enzyme. The electrophoresis was performed using a CHEF DRII system (Bio-Rad, Marnes-la-Coquette, France) and the conditions consisted of an initial time of 2.2 s, a final time of 54.2 s at a gradient of 6 V cm^−1^ and an included angle of 120°. The gels were electrophoresed for 24 h. *Salmonella enterica* serotype Braenderup strain H9812 (ATCC BAA664, Manassas, VA, USA) was used as a molecular weight standard. A dendrogram was constructed using the Dice similarity coefficient and the unweighted-pair group method by average linkages (UPGMA) or neighbor joining algorithm with 3 % position tolerance using Bionumerics 4.0 software (Applied Maths, Austin, Texas, USA).

### Biofilm assay

Qualitative biofilm assay was performed following the procedure described previously [27]. Briefly, overnight bacterial cultures were diluted to 1:100 in M9 minimal medium (BD Technologies) containing 10 μg/ml niacin or LB medium followed by inoculation into U-bottom 96-well plates (Denville) in triplicates. The plates were incubated at room temperature for 24 h at 37 °C. The plates were washed three times with distilled water and biofilms were stained with 0.1 % crystal violet for 15 min. After three washes with distilled water, the presences or absence of biofilms was evaluated.

### Antibiotic susceptibility testing

Kirby-Bauer (KB) disk diffusion assay was conducted to determine antibiotic resistance profiles using antibiotic paper disks (BD Technologies). The antibiotics used in KB test were amikacin (30 μg), amoxicillin with clavulanic acid (20/10 μg), chloramphenicol (30 μg), kanamycin (30 μg), nalidixic acid (30 μg), sulfamethoxazole with trimethoprim (23.75/1.25 μg), sulfisoxazole (0.25 μg), and tetracycline (30 μg). Susceptibility or resistance profiles were interpreted according to Clinical Laboratory Standards Institute (CLSI) guidelines. Extended spectrum β-lactamase (ESBL) sensitivity profiles were screened using confirmatory ESBL plates according to manufacturer’s instructions (Sensititre; Thermo Fisher Scientific, Oakwood Village, OH, USA). The dilution range of antibiotic concentrations used were as follows; cefazolin (8–16 μg/ml), cefepime (1–16 μg/ml), cefoxitin (4–64 μg/ml), meropenem (1–8 μg/ml), cephalothin (8–16 μg/ml), cefpodoxime (0.5–64 μg/ml), ceftriaxone (1–128 μg/ml), ciprofloxacin (1–2 μg/ml), gentamicin (4–16 μg/ml), ampicillin (8–16 μg/ml), imipenem (0.5–16 μg/ml), piperacillin/tazobactam (4/4–64/4 μg/ml), ceftazidime (0.25–128 μg/ml), ceftazidime/clavulanic Acid (0.25/4–128/4 l μg/ml) and cefotaxime (0.25–64 μg/ml). Plates were incubated at 37 °C for 18 h and read automatically using Sensititre System (Thermo Fisher Scientific).

### Multilocus sequence typing

Multilocus sequence typing (MLST) of ESBL-positive *E. coli* isolates was performed using seven housekeeping genes (*adk*, *fumC*, *gyrB*, *icd*, *mdh*, *purA*, and *recA*), as described previously [49]. Primer sequences were obtained from the *E. coli* MLST database website (http://mlst.ucc.ie/mlst/dbs/Ecoli) (Table 7). The cycling conditions were as follows: initial denaturation at 95 °C for 5 min, followed by 30 cycles of initial denaturation at 95 °C for 30 s, primer annealing at the melting temperatures for 30 s, and extension at 72 °C 1 min and followed by a final extension at 72 °C for 10 min. The PCR products were treated with exonuclease I and shrimp alkaline phosphatase (Affymetrix, Santa Clara, CA, USA) and sequenced at the Genomics Core Facility (Penn State University) with the same primers used to generate PCR products. Sequences were compared with *E. coli* MLST database website to determine the MLST types.

### In-vitro cell invasion assay

The hCMEC/D3 cell line, which is known to possess the main characteristics of primary brain endothelial cells, was kindly provided by Dr. Babette Weksler at Weill Cornell Medical College (New York, NY, USA) [50]. Invasion assays were performed in triplicates using the method described previously [51]. Briefly, hCMEC/D3 cells were seeded on 96-well tissue culture plates coated with rat collagen (R&D Systems, Inc., Minneapolis, MN, USA) and maintained at 37 °C for six days in a humidified chamber containing 5 % CO_2_ to form a confluent monolayer. The complete medium used for culturing of hCMEC/D3 cells was EBM-2 endothelial basal medium (Lonza, Allendale, NJ, USA) containing 5 % bovine serum (GE Healthcare Bio-Sciences, Pittsburgh, PA, USA), 1.4 μM hydrocortisone (Sigma-Aldrich, St. Louis, MO, USA), 5 μg/ml ascorbic acid (Sigma), 1 % chemically defined lipid concentrate (Invitrogen, Carlsbad, CA, USA), 10 mM HEPES (GE Healthcare Bio-Sciences), and 1 ng/ml human basic fibroblast growth factor (Sigma). Cells were infected with *E. coli* strains with a multiplicity of infection (MOI) of 100 (100 bacteria per hCMEC/D3) and incubated for 2 hours at 37 °C in 5 % CO_2_. Cells were then washed with phosphate-buffered saline (PBS, pH 7) three times and incubated with fresh complete EBM-2 medium containing 100 μg/ ml gentamicin (Sigma) for one hour to kill extracellular bacteria. Subsequently, 0.2 ml of 0.01 % Triton X-100 (Sigma) was added to each well to release intracellular bacteria. Cells were fully disrupted by repeated pipetting and viable bacteria were enumerated by plating onto LB agar. Invasion frequencies were calculated comparing the ratio between the number of colony forming units (CFU) released from the cells and the number of CFU inoculated per well. To standardize the comparison of invasion frequencies, relative invasion was calculated as a percent of invasion in comparison to the well-characterized NMEC strain RS218, which was arbitrarily set at 100 %.

## Ethics statement

Both NMEC and HFEC isolates, in their entirety, were collected for purposes other than this study and were given without any Health Insurance Portability and Accountability Act (HIPAA) identifiers by Dr. K.S. Kim (Johns Hopkins University, Baltimore, MD, USA) and Dr. C. DebRoy (*E. coli* Reference Center, University Park, PA, USA).

### Statistical analysis

Data were statistically analyzed by Fisher’s exact test (virulence genotyping and phylogrouping), two-tailed student’s t -test (invasion frequency), and a *p* < 0.05 was considered statistically significant. The heat map of virulence gene profiles was constructed using the R code (version 3.1.1).

## Availability of supporting data

The raw data supporting the results of this article are provided in the Additional file 1: Table S1.

## Additional file

Additional file 1: Table S1. Distribution of phylogroups, virulence genes, ability to form biofilms, and relative invasion frequencies of NMEC and HFEC isolates. Detailed procedure for each experiment is included in the Materials and Methods section. Raw data of phylogroups, virulence genes, ability to form biofilms, and invasion frequencies observed with in vitro invasion assays for *E. coli* isolates used in the study are summarized in Additional file 1: Table S1. (XLSX 27 kb)

## Abbreviations

BBB: Blood brain barrier
CFU: Colony forming units
CLSI: Clinical Laboratory Standards Institute
ESBL: Extended spectrum β-lactamase
ExPEC: Extra-intestinal pathogenic *E. coli*
hCMEC: Human cerebral microvascular endothelial cells
HEPES: 4-(2-hydroxyethyl)-1-piperazineethanesulfonic acid
HFEC: Healthy fecal *E. coli*
MLST: Multi-locus sequence typing
NMEC: Neonatal meningitis-causing *E. coli*
PBS: Phosphate buffered saline
PCR: Polymerase chain reaction
PFGE: Pulsed-field gel electrophoresis
UPEC: Uropathogenic *E. coli*
VFs: Virulence factors
